# Viridans Group Streptococcal Infections in Children After Chemotherapy or Stem Cell Transplantation: A 10-year Review From a Tertiary Pediatric Hospital

**DOI:** 10.1097/MD.0000000000002952

**Published:** 2016-03-07

**Authors:** Maryke J. Nielsen, Sarah Claxton, Barry Pizer, Steven Lane, Richard P.D. Cooke, Stéphane Paulus, Enitan D. Carrol

**Affiliations:** From the Department of Oncology, Alder Hey Children's NHS Foundation Trust (MJN, BP); Institute of Infection and Global Health, University of Liverpool (SC, EDC, SP); Institute of Translational Medicine, University of Liverpool (SL, BP); Department of Microbiology, Alder Hey Children's NHS Foundation Trust (RPDC); and Department of Infectious Diseases, Alder Hey Children's NHS Foundation Trust (SP, EDC), Liverpool, UK.

## Abstract

Viridans Group Streptococci (VGS) are associated with high mortality rates in febrile neutropenia; yet there are no recent European pediatric studies to inform antimicrobial therapy. The aim of this study was to describe the characteristics, outcome, and resistance patterns of children with VGS bacteremia (VGSB) undergoing treatment of malignancy or hematopoietic stem cell transplant.

Patients aged 0 to 18 years, admitted to a tertiary pediatric hemato-oncology center with VGSB, from 2003 to 2013, were included in the study. All data were collected retrospectively from medical records.

A total of 54 bacteremic episodes occurred in 46 patients. The most common underlying diagnosis was relapsed acute lymphoblastic leukemia.

*Streptococcus mitis* was the most frequent organism. A total of 30% of isolates were resistant to penicillin and 100% sensitive to vancomycin. There were 8 episodes (14.8%) of Viridans Group Streptococcal Shock Syndrome; 6 resulted in admission to intensive care and 3 of these patients died of multiorgan failure.

The potentially fatal nature of VGSB is confirmed. The high risk in relapsed acute lymphoblastic leukemia is of note. Research is needed to develop risk-stratification scores that identify children at risk of Viridans Group Streptococcal Shock Syndrome to guide empirical antimicrobial therapy in febrile neutropenia.

## INTRODUCTION

Viridans Group Streptococci (VGS) are increasingly recognized as both a frequent and life-threatening cause of infection in children and adults immunocompromised as a result of chemotherapy or hemopoietic stem cell transplant (HSCT). Though part of the normal flora of the human gastrointestinal and genital tracts, VGS are now the third most common cause of bacteremia in pediatric hematology and cancer patients globally.^1^ VGS bacteremia (VGSB) may result in the development of Viridans Group Streptococcal Shock Syndrome (VSSS), a toxic shock-like syndrome, characterized by hypotension and/or acute respiratory distress syndrome (ARDS).^2,3^ VSSS is associated with reported rates of intensive care unit (ICU) admission of up to 64%^4^ and mortality rates of 0% to 23%.^3,5–8^ Predisposing factors linked to the development of VGSB are well described and include acute myeloid leukemia (AML), HSCT, high-dose cytarabine, severe mucositis, prolonged fever, prolonged neutropenia, and pneumonia.^6,7,9^

International guidelines both in pediatric and adult practice recommend consideration of empirical glycopeptides for patients who are clinically unstable, in whom there is a high suspicion of penicillin resistance,^10,11^ or for specific clinical indications including catheter-related infection, skin or soft tissue infection, and pneumonia.^11^ Rising rates of in vitro penicillin resistance amongst VGS isolates, of 21% to 62%,^12^ have encouraged the use of empirical glycopeptides in first-line management of febrile neutropenia, with usage rates of up to 96% in some adult centers.^13^ A single-center adult study found penicillin resistance in VGSB in neutropenic patients to be associated with the presence of at least one of the following risk factors in 98% of cases: β-lactam prophylaxis, receipt of a β-lactam in the previous 30 days, and nosocomial onset of infection. Limiting empirical antigram positive therapy to neutropenic patients with at least one of these variables was predicted to reduce such use by 42%.^13^ These risk factors have not been investigated in other settings or in pediatrics.

There is a paucity of information regarding outcomes of VGSB in pediatric oncology in the United Kingdom and the rest of Europe, the frequency of VSSS, rates of VGS penicillin resistance, and antimicrobial prescribing practice. The aim of this study was to establish the clinical characteristics, management, outcomes, and resistance profiles of children with VGSB undergoing treatment of malignancy in a tertiary referral center in the United Kingdom.

## METHODS

A computer-based search of our microbiology laboratory database was conducted to identify all VGS blood culture isolates from 2003 to 2013 in a pediatric oncology/hematology unit at a tertiary pediatric hospital in Liverpool, United Kingdom. All patients aged 0 to 18 years currently receiving chemotherapy for hematological or solid organ malignancy or undergoing HSCT at the time of positive culture were included in the study.

Episodes of blood culture-positive VGS which failed to meet CDC criteria for either device-associated laboratory-confirmed bloodstream infection (LCBI) or mucosal barrier injury laboratory-confirmed bloodstream infection (MBI-LCBI) were excluded from the study.^14^ Patients with an episode of VGSB during HSCT for a nonmalignant condition were included in the study, although VGSB associated with neutropenia in nonmalignant conditions outside of HSCT were excluded.

Neutropenia was defined at an absolute neutrophil count <0.5 × 10^9^/L. VSSS was defined as hypotension or other evidence of inadequate cardiac output requiring intravascular volume expansion or inotropic support and/or respiratory insufficiency, presenting as ARDS necessitating assisted ventilation,^8^ with documented evidence of VGSB.

Antimicrobial resistance testing was carried using the disc diffusion method according to guidelines from the British Society for Antimicrobial Chemotherapy. Penicillin resistance was defined as minimum inhibitory concentration (MIC) breakpoint of >2 mg/L, intermediate sensitivity an MIC 0.5 to 2 mg/L, and sensitive as ≤0.25 mg/L. Glycopeptide sensitivity was defined as MIC breakpoint ≤2 mg/L and resistance as MIC >2 mg/L.

Over the 10 years of the study, empiric management of febrile neutropenia changed from ceftazidime and amikacin at the end of 2006, to piperacillin/tazobactam + gentamicin, and then finally to the current protocol of piperacillin/tazobactam alone in December 2012. All data were collected retrospectively from both electronic and paper-based medical records by authors MJN and SC. Statistical analysis was carried out using SPSS 21. All tests for significance were 2-sided and statistical significance defined as *P* < 0.05. To allow for some patients (n = 5) having multiple episodes (n = 13), a multilevel logistic regression model was used to examine the relationship between the clinical variables and penicillin resistance. The multilevel model adjusted the analysis to allow for within-patient clustering of episodes.

As a retrospective review of routine clinical practice, antimicrobial resistance, and subsequent outcomes, ethical approval was deemed not necessary; however, approval from the Director of Research was given under the hospital's remit of service evaluation.

## RESULTS

**FIGURE 1:**
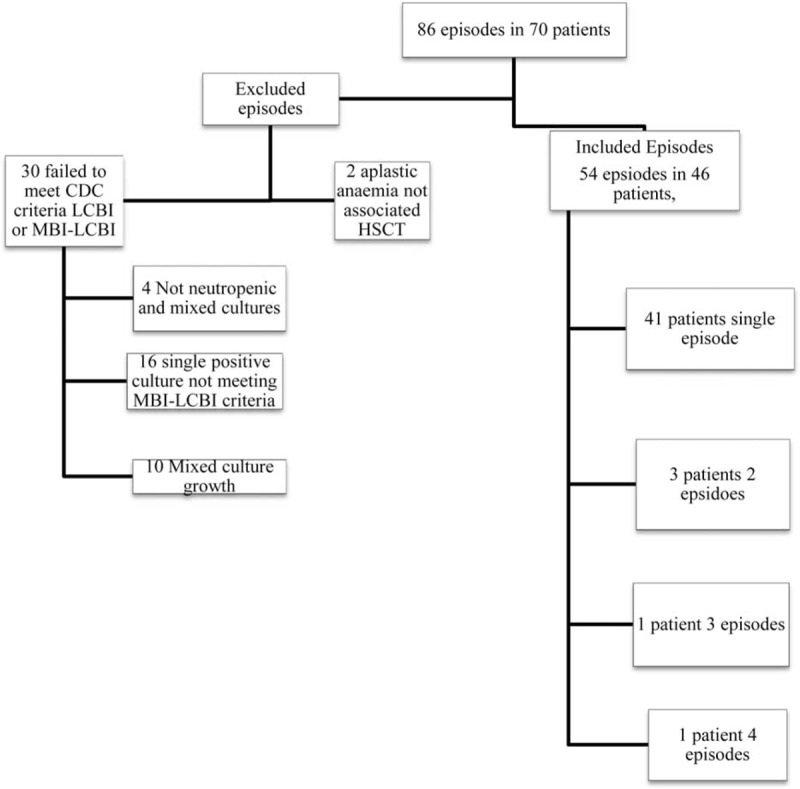
Consort statement.

The search initially identified 86 episodes of VGSB in 70 individual patients. Fifty-four discrete episodes of VGS bloodstream infections, occurring in 46 patients, met inclusion criteria (Figure 1). One to three positive blood cultures were taken during a single blood draw for a single episode due to the practice of sampling each lumen of a central venous access device. No patients were lost to follow-up and data were available for all patients included in the study.

Median patient age was 9 years (interquartile range [IQR] 3–13 years), with 24 episodes occurring in females and 30 in males. Hematological and oncological diagnoses are shown in Table 1. The most common diagnoses were relapsed acute lymphoblastic leukemia, acute lymphoblastic leukemia (ALL), and acute myeloid leukemia (AML). VGS species isolated were *Streptococcus mitis* (35 episodes), *S oralis* (10 episodes), *S mitis* and *S oralis* (3 episodes), *S salivarius* (3 episodes), *S viridans* (2 episodes unspeciated), and *S sanguinis/gordonii* (1 episode).

**TABLE 1:**
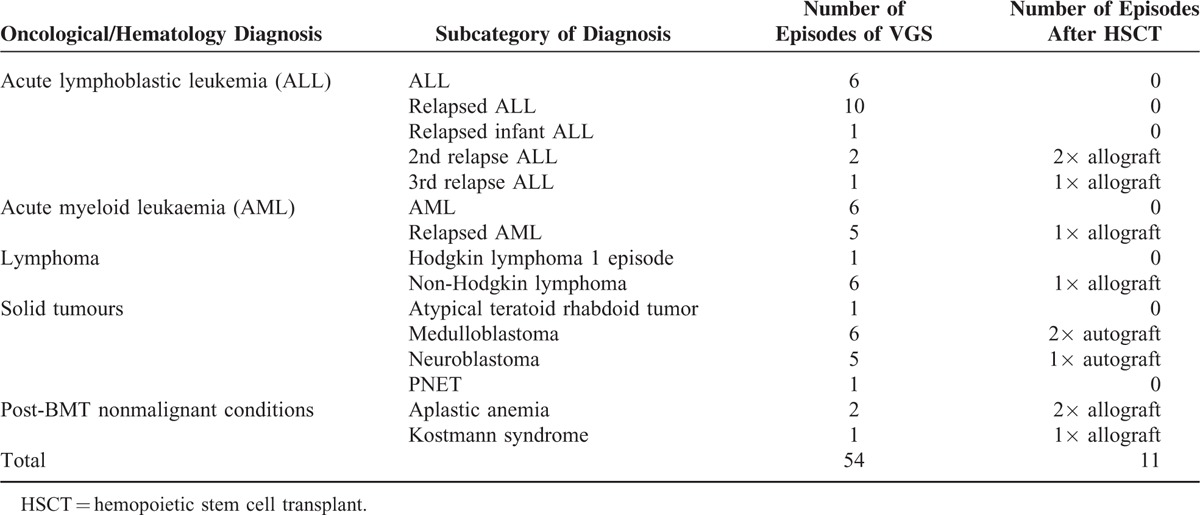
Hematological or Oncological Diagnosis of Patients and Number Treated With Hemopoietic Stem Cell Transplant (HSCT)

### Clinical Symptoms

Mucositis was present in 42.6% of patient episodes (23/54). Median temperature at presentation was 38.3°C (IQR 38–38.7°C). Fever lasted for a median duration of 5 days from the day of positive cultures (IQR 2–10 days). Symptoms at presentation, other than fever, included cough, diarrhoea and vomiting, coryzal symptoms, chest pain, dyspnea, and pharyngitis. In 14/54 episodes, the only presenting symptom was fever. In 3 episodes, a gastrointestinal bleed occurred at presentation, and in 1 episode, pulmonary hemorrhage was present. Skin/soft tissue infection was present in 1 patient. Pneumonia was diagnosed in 10 patients, of whom 6 developed VSSS. Three patients underwent dental extraction in the 4 weeks preceding the episode of VGS. Duration of neutropenia from day of positive culture ranged from 0 to 56 days (median 10 days; IQR 5.8–16.3 days). Fifty per cent of episodes were classified as nosocomial in onset (occurring 48 hours or more into hospital stay). All but 2 patients had a central venous catheter in situ during the episode of VGSB, most frequently a double lumen broviac catheter. No patient required removal of the central venous catheter due to VGSB.

### Chemotherapy

Ten episodes (18.5%) of VGSB occurred in the immediate posttransplant phase: 7 after allograft and 3 episodes after autograft. A further single episode occurred in a patient with graft versus host disease 3 months after allogenic bone marrow transplant for EBV lymphoma, cartilage-hair cell hypoplasia, and T-cell immunodeficiency. For those episodes after chemotherapy, the preceding course included either an anthracycline or cytarabine in 26 episodes. This was an anthracycline alone in 10/43 of episodes, most commonly danuorubicin, or cytarabine in 10 episodes and both drugs in 6 episodes.

### Viridans Streptooccal Shock Syndrome and Patient Outcomes

Of the 54 episodes of VGSB in the cohort, VSSS developed in 8 episodes (14.8%). Characteristics of patients with VSSS are shown in Table 2. Three patients developed ARDS only, 3 presented with shock and ARDS, and 2 presented with shock alone. Six of the VSSS patients were admitted to the ICU and 3 patients subsequently died. Two of the patients who died from VSSS were postallogenic transplant, aged 16 and 18 years, for aplastic anemia and relapsed ALL, respectively. The third patient was a 3-year-old with trisomy 21, who developed VGS after a cycle of chemotherapy for AML. All 3 of these patients were admitted to the ICU with ARDS, subsequently developed multiorgan failure, and died on ICU between 15 and 20 days after admission.

**TABLE 2:**
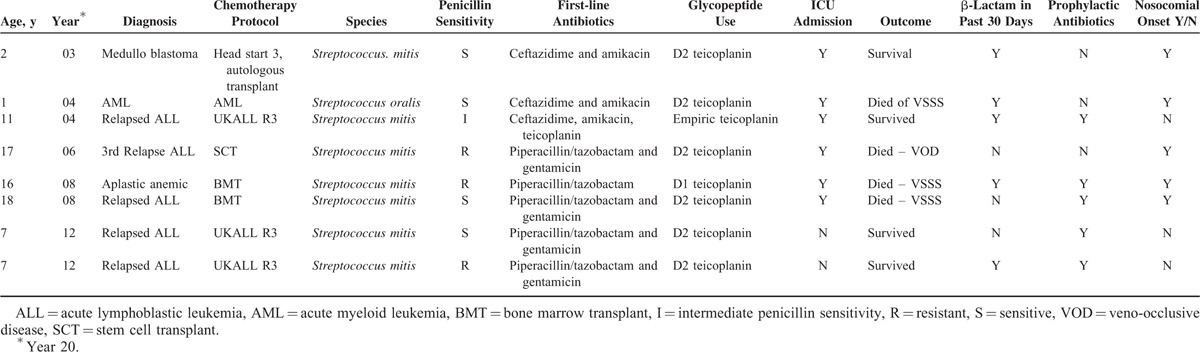
Characteristics of Patients With Viridans Streptococcal Shock Syndrome (VSSS)

One patient with VSSS subsequently died due to veno-occlusive disease not thought to be related to sepsis during the same admission. Two other patients who did not develop VSSS required management on the ICU during the same admission as VGSB but for unrelated problems; 1 for candidemia and enterococcal sepsis, and the second 2 months after VGSB sepsis with pneumonia, renal failure, and hemorrhagic cystitis. Fifty per cent of VGS isolates in VSSS were fully susceptible to penicillin. The overall mortality rate for VGSB in this cohort was 5.6%.

### Antibiotic Management and Susceptibility Profiles

Treatment with a glycopeptide was given in over 98% (53/54) of cases; however, only 3 patients received empirical glycopeptide therapy at the start of treatment (teicoplanin on all occasions). A glycopeptide was commenced on a mean of day 2 (range 1–4 days), after initial blood culture results. Teicoplanin was the first-line agent in 48 episodes and vancomycin in 5 episodes. Teicoplanin was escalated to vancomycin in 11 episodes in response to clinical deterioration. One patient's antimicrobial therapy was stepped down from vancomycin to teicoplanin. There were no episodes in which blood cultures remained positive for VGS after commencement of antimicrobial therapy.

Viridans Group Streptococci resistant to penicillin were cultured in 29.6% of episodes with a further 7.4% of episodes due to a VGS of intermediate penicillin sensitivity. Isolates were universally tested for susceptibility to vancomycin, and 100% were found to be sensitive. Of the 48 patient episodes in which teicoplanin sensitivity was tested, 100% of VGS were found to be sensitive. Episodes with penicillin-sensitive and nonpenicillin-sensitive isolates are compared in Table 3.

**TABLE 3:**
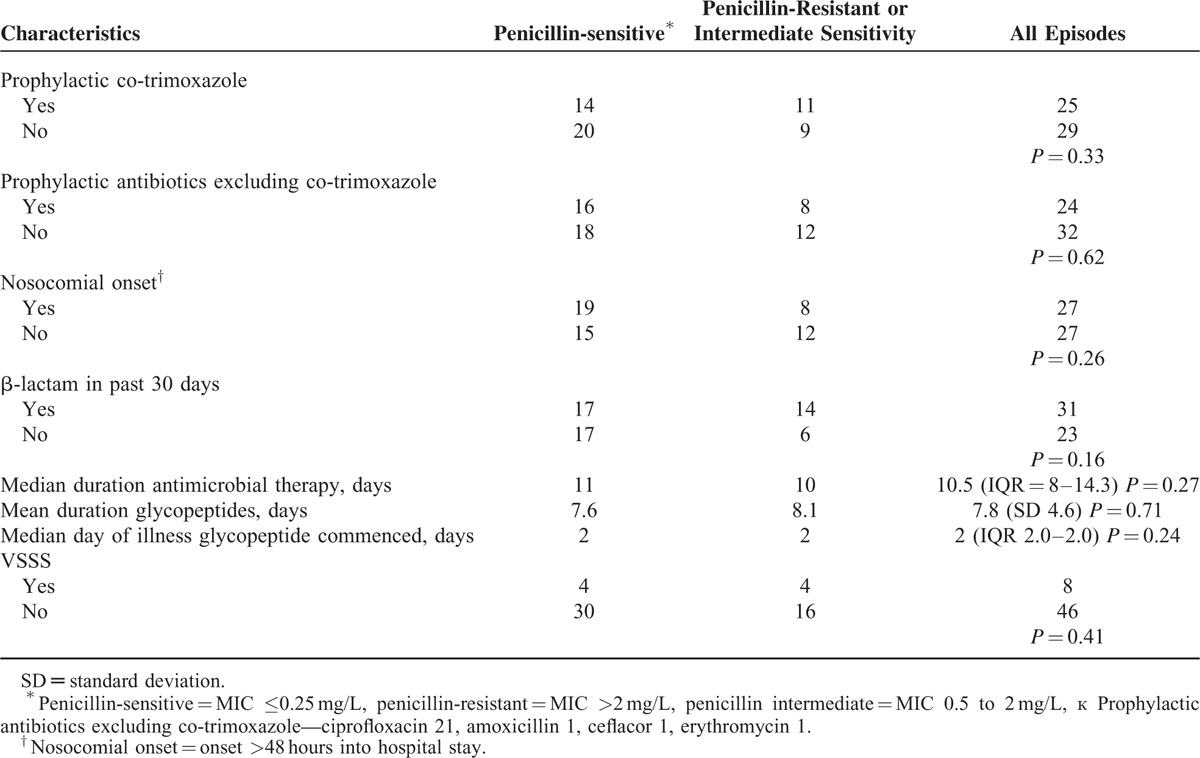
Comparison of Penicillin-sensitive and Nonsensitive Patient Episodes

## DISCUSSION

This is the first study in a decade characterizing VGSB in pediatric cancer patients in Europe. Fifteen per cent of VGSB resulted in VSSS, which is equal to the rate of VSSS from the last British study,^2^ a single-center descriptive study conducted from 1995 to 1999. The mortality rate directly attributable to VGSB of 5.6% is similar to recent data both from oncology units from the rest of the world of 1.8% to 7%,^6–8^ and from larger population-based studies examining VGS in AML.^9^ Research from centers with lower mortality rates excludes high-risk patients such as those undergoing HSCT,^4^ relapsed patients,^5^ high-risk chemotherapy regimens, or those with comorbidities such as Down syndrome.^15^

The global rise of VGSB in hemato-oncology patients is thought to be due to the increasing use of indwelling central venous catheters, increasingly intense antineoplastic therapy causing severe mucositis, and the widespread use of empiric broad-spectrum antibiotics.^16^ The trend is in keeping with a shift towards gram-positive organisms as the predominant organisms in febrile neutropenia accompanied by rising levels of antimicrobial resistance in the community and hospital settings.^17^ The most recent MRC AML protocol has also been associated with VGS infection.^9^

In line with known risk factors for VGSB, patients in this cohort had frequently recently received chemotherapy featuring cytarabine and anthracyclines, both highly myelotoxic drugs known to cause prolonged severe neutropenia and severe mucositis. Mucositis was present in 42% of patients. It is unclear if mucositis is a risk factor independent of the chemotherapy regimens used. The major portals of entry for VGSB into the bloodstream are probably damaged oral and gastrointestinal mucosa.^12,16^ Low incidence of VGSB in AML in some centers has been attributed to effective mouth and gut decontamination.^5^

The most frequent hematological or oncological diagnosis was relapsed ALL, whereas previous studies have found AML the most common hemato-oncology diagnosis in VGSB patients.^6,8^ The Relapsed ALL R3 protocol was introduced in 2003; patients most frequently developed VGS during weeks 7 to 11 of the protocol. Clinicians should therefore be vigilant for the development of VGSB when patients are being treated during these weeks of this protocol. Similarly, a high index of suspicious should be exercised in patients post-HSCT as, a fifth of cases occurred in the posttransplant period.

Rates of penicillin resistance at 29.6% are lower than the 2 prior European pediatric studies of 67.5%^2^ and 36% to 45%,^18^ and those reported in more recent studies from the rest of the world at 40.7% to 70%.^3,6^ Use of empiric glycopeptide is also lower than that used in other studies.^6,13^ This is accounted for by a rigorous antimicrobial stewardship program, and a febrile neutropenia antibiotic prescribing policy that follows guidelines from the National Institute of Clinical Excellence,^9^ agreed by both the infectious disease and hemato-oncology team, which does not advocate routine use of glycopeptides empirically. There is an absence in the pediatric literature on treatment of VGSB and vancomycin-related toxicity, which is surprising, given the controversy regarding monitoring and dosing of vancomycin in children and the high rates used in some centers.

No significant difference was found between fully penicillin-sensitive VGS isolates and those resistant to penicillin and/or of intermediate sensitivity in relation to nosocomial onset of sepsis, use of prophylactic antibiotics, or receipt of β-lactam therapy within the preceding 30 days, which is in contrast to a recent large adult study.^13^ There was also no difference in the number of cases of VSSS between these 2 groups. Failure to demonstrate significant differences between these 2 groups may be due to the small sample size particularly compared with adult studies. In the absence of pediatric factors known to be predictive of penicillin resistance in VGSB, there is limited evidence available to inform targeted empirical glycopeptide use.

International guidelines such as that issued by the UK National Institute of Clinical Excellence (NICE), the Infectious Disease Society of America (IDSA), European Conference on Infections in Leukaemia 2011, and the Canadian Paediatric Haematology/Oncology Network recommend risk stratification of febrile neutropenia into high and low risk.^10,11,19,20^ Yet, risk-based scoring systems are better at identifying those patients at low risk of complications rather than those at high risk,^21,22^ and reference few of the known risk factors for VGS. Identification of patients who will develop VSSS is known to be challenging. A single retrospective pediatric study has identified factors predictive of VSSS, finding that peak temperature at presentation in the range 39.1 to 39.7 and in-patient status to be significant,^8^ whereas a population-based cohort study of children with AML did not identify any factors predictive of VSSS.^9^ Large multicenter studies are needed to develop and validate rapid risk-stratification scores that can identify patients at high risk of VSSS and other adverse outcomes from febrile neutropenia, providing more personalized empiric management of febrile neutropenia.

A limitation to this single-center study is that many children in the region served by the study hospital attend district “shared care” hospitals rather than the tertiary center for management of uncomplicated episodes of febrile neutropenia and hence there is a bias towards more high-risk patients in this cohort. Considerable heterogeneity exists within the literature regarding the definition of VGSB in children with cancer. This is the first pediatric study to our knowledge utilizing the recently introduced CDC criteria for MBI-LCBI. Controversy exists as to whether these new definitions accurately identify catheter-related bloodstream infections.^23^ The strict application of these guidelines as inclusion/exclusion criteria will have resulted in the exclusion of cases in nonneutropenic patients which are included in other studies and inclusion of some that would have been excluded in previous studies on the grounds of a single positive culture only.

In conclusion, this study has confirmed the potentially fatal nature of VGSB in children undergoing chemotherapy and HSCT. Significant rates of penicillin resistance in VGS were also found. Patient characteristics were in keeping with known risk factors for VGS, but with a high prevalence of patients receiving treatment for relapsed ALL on the R3 protocol, an observation that has not previously been noted and requires further research.
